# A. Fells

**Published:** 1939

**Authors:** 


### ARTHUR FELLS, M.B., C.M., F.R.C.S.E.

The death occurred suddenly on 9th December, 1939, while on his way to see a patient, of Dr. Arthur Fells, of 19 Alexandra Road, Clifton.

Dr. Fells, who was 70 years of age, had been in practice in Bristol for thirty-four years. He had been surgeon to the Bristol Eye Dispensary, surgeon to the out-patient department, Bristol Children's Hospital, and medical registrar to the Bristol General Hospital.

A native of Kent, Dr. Arthur Fells took his degrees of M.B., C.M., F.R.C.S., at Edinburgh, and went to India, where he was surgeon to the London Missionary Society Hospital at Neyoor, remaining for thirteen years.

He then came to practice in Bristol, and during the Great War was surgeon to the hospital which was opened at the Red Maids' School.

He was also Acting Honorary Surgeon at the Royal Infirmary at a time during the Great War when many of the surgeons of that Institution were serving overseas.

Dr. Arthur Fells leaves a widow, two sons and a daughter, to whom we offer our sincere sympathy.

